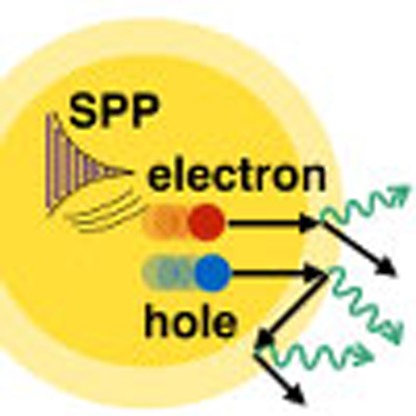# Theory and computation of hot carriers generated by surface plasmon polaritons in noble metals

**DOI:** 10.1038/ncomms8044

**Published:** 2015-06-02

**Authors:** Marco Bernardi, Jamal Mustafa, Jeffrey B. Neaton, Steven G. Louie

**Affiliations:** 1 Department of Physics, University of California at Berkeley, 366 LeConte Hall #7300, Berkeley, California 94720, USA; 2 Materials Sciences Division, Lawrence Berkeley National Laboratory, Berkeley, California 94720, USA; 3 Molecular Foundry, Lawrence Berkeley National Laboratory, Berkeley, California 94720, USA; 4 Kavli Institute for Energy Nanosciences at Berkeley, Berkeley, California 94720, USA

## Abstract

Hot carriers (HC) generated by surface plasmon polaritons (SPPs) in noble metals are promising for application in optoelectronics, plasmonics and renewable energy. However, existing models fail to explain key quantitative details of SPP-to-HC conversion experiments. Here we develop a quantum mechanical framework and apply first-principles calculations to study the energy distribution and scattering processes of HCs generated by SPPs in Au and Ag. We find that the relative positions of the *s* and *d* bands of noble metals regulate the energy distribution and mean free path of the HCs, and that the electron–phonon interaction controls HC energy loss and transport. Our results prescribe optimal conditions for HC generation and extraction, and invalidate previously employed free-electron-like models. Our work combines density functional theory, GW and electron–phonon calculations to provide microscopic insight into HC generation and ultrafast dynamics in noble metals.

## Abstract (web summary)

Hot carriers generated by surface plasmons in metal structures are garnering interest for their use in optoelectronics or photocatalysis. Bernardi *et al*. present a quantum mechanical framework to study their properties and find the optimalyconditions for their generation and extraction.

Surface plasmon polaritons (SPPs) are electron collective excitations generated by light at the interface between a metal and a dielectric^1^. Modelling of SPPs is dominated by approaches that employ classical electromagnetism and account empirically for the properties of the materials supporting the SPP. Yet, the inherently quantum mechanical nature of SPPs^2^,^3^ becomes manifest in their scattering and decay processes in bulk and nanoscale materials. A key example is the decay of SPPs to electron-hole pairs, a process whose crucial importance is twofold: first, it is a main energy loss mechanism of SPPs in metals, currently limiting the applicability of plasmonics^4^,^5^; second, it leads to generation of hot electrons and holes with application in several branches of applied physics^6^. Recent experiments reported the extraction of such SPP-induced hot carriers (HCs) in metals before they thermalize—typically over nanometer lengths and sub-picosecond times—by injecting the HCs over a Schottky barrier into a semiconductor or oxide^7^,^8^,^9^, or by transferring them into surface adsorbates to perform chemical reactions with large activation barriers^6^,^10^,^11^. The vast literature on HCs in noble metals generated by intense light pulses^12^,^13^,^14^ (instead of SPPs), together with recent calculations on SPP-to-HC conversion^15^,^16^, help interpret these recent experiments on SPP-induced HCs.

Lack of predictive and accurate quantum mechanical approaches to study HCs generated by SPPs or photons has led to ambiguity in the microscopic interpretation of experiments involving HCs in Au and Ag, two mainstay materials in plasmonics^4^. In particular, recent studies indicate that SPP decay excites electronic transitions from occupied states close to the Fermi energy (*E*_F_)^10^, implying that most of the SPP energy goes into hot electrons rather than hot holes, and that nanocrystals with <10 nm diameter are necessary to obtain HCs with significant energy^17^ (for example, 1–2 eV away from *E*_F_). The mean free paths (MFPs) of HCs in noble metals appear to be short^10^, though a quantitative estimate of the MFPs and their dependence on crystal direction is not available. In addition, it is often assumed that HC scattering and thermalization are dominated by Auger processes^10^,^14^, since thermalization induced by phonons occurs on a slower time scale^14^. As shown in this work, all these conclusions must be revised.

Here, we develop a quantum mechanical framework to study the energy distribution of HCs generated by SPPs and photons in Au and Ag, and employ *ab initio* calculations of the electron–phonon (*e*–ph) and electron-electron (*e*–*e*) interactions to study the MFPs and relaxation times of HCs within 5 eV of the Fermi energy. Our approach is free of experimental input, and combines density functional theory (DFT)^18^, the GW (where G is the Green function, W is the screened Coulomb potential, and GW is the diagram employed for the electron self energy) method^19^, and *ab initio e*–ph calculations^20^. Our ability to use extremely fine grids for Brillouin zone (BZ) integrations allows us to resolve HC scattering with unprecedented accuracy^21^. We find that the interband transition threshold (between *d* and *s* states) defines two regimes for HC generation and transport. The decay of SPPs with energy lower than the interband threshold leads to generation of long-lived HCs with long isotropic MFPs of up to 40 nm and energy within 1–2 eV of *E*_F_. On the other hand, decay of SPPs with energy higher than the interband threshold leads to generation of short-lived hot holes in *d* states with anisotropic and short (sub 5 nm) MFPs, and hot electrons with only <1 eV energy above *E*_F_. The regime characterized by SPP energy below the interband threshold is better suited to employ HCs in applications requiring long MFPs, and allows one to optimize HC generation by tuning the SPP energy. These results represent an important first step to understand SPP decay and energy loss, and to control HC generation and transport in bulk and nanostructured noble metals.

## Results

### Theory of SPP—electron coupling

Coupling of SPPs or photons to electrons in materials can be described in the framework of many-body perturbation theory^22^. The lowest order Feynman diagram for this coupling process, shown in Table 1, describes a boson (here, SPP or photon) coupling to the electron gas through the electronic polarizability *χ* and a coupling matrix element *g*. This diagram is analogous to the lowest order coupling of phonons to electrons^23^,^24^. The decay rate Γ(**q**_p_,*E*_p_) for a boson of momentum *ħ***q**_p_ and energy *E*_P_ to electron–hole pairs is proportional to the imaginary part of the self-energy^22^:


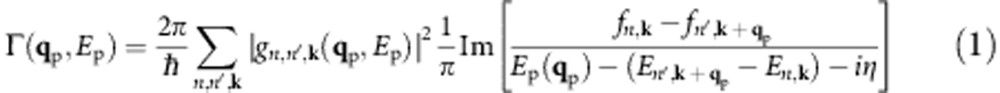


where −*E*_*n*,**k**_ and 
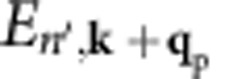
 are, respectively, the quasiparticle energies of the hot hole in a state with band *n* and crystal momentum –**k**, and hot electron in a state with band *n*′ and crystal momentum **k**+**q**_p_, as generated in the boson decay process. In addition, *f*_*n*,**k**_ and 
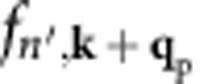
 are Fermi occupations, *η* is a small broadening, and the sum is contributed by electronic transitions from occupied to empty states that differ by *ħ***q**_p_ in crystal momentum and *E*_p_ in energy. If the matrix elements have a weak band and **k** dependence, the decay rate of a SPP or photon is proportional to the imaginary part of the electronic polarizability, Im*χ*(**q**_p_,*E*_P_). 

Here, we employ a quantity proportional to Im*χ*(**q**_p_,*E*_P_), the finite momentum joint density of states (FM-JDOS) *J*(**q**_p_,*E*_P_), defined as the number of states per unit energy separated by momentum *ħ***q**_p_ and energy *E*_p_ in the quasiparticle bandstructure:


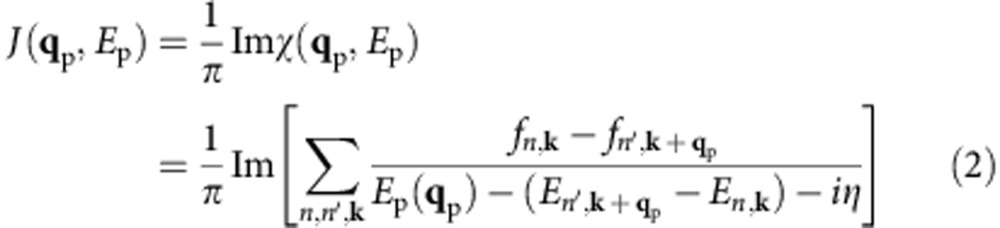


The FM-JDOS is a measure of the phase space available for the decay of a SPP or photon with momentum *ħ***q**_p_ and energy *E*_p_. Greater FM-JDOS values generally would correspond to greater SPP decay and HC generation rates.

The coupling matrix elements in equation (1) can be derived with a range of theoretical approaches. We first discuss the coupling of electrons to plasmons—that is, collective electronic excitations in the absence of light—since theories developed for plasmons have been occasionally applied to SPPs^25^. The so-called Landau damping^26^ treatment of the electron gas derives the decay rate Γ_p_ of a bulk plasmon in the weak coupling limit via the zeros of the complex macroscopic dielectric function *ɛ*_M_ of the metal close to the real energy axis, that is, 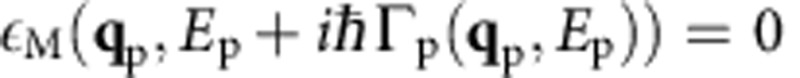. Due to the bosonic nature of plasmons, the decay rate Γ(**q**_p_,*E*_p_) has the form given in equation (1), with coupling matrix elements^26^ 
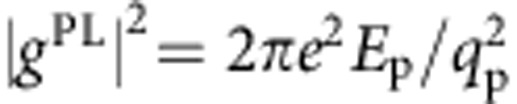
. In spite of its common use, Landau damping is rarely discussed in terms of the diagram in Table 1 and the coupling *g*^PL^, though this viewpoint elucidates its physical origin.

The coupling matrix elements for SPPs and photons can be derived by second quantization of the vector potential^27^ (see Methods). For photons, the coupling is given by the well-known dipole matrix element 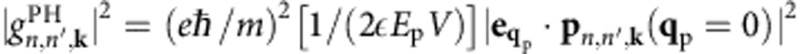, where *V* is the volume, 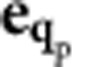 is the polarization vector, and 
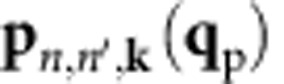
 the transition dipole matrix element. For SPPs at a metal–dielectric interface, we apply a vector potential second quantization procedure^28^,^29^ (see Methods) to obtain the SPP–electron coupling 
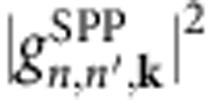
 given in Table 1. The SPP–electron coupling has a form analogous to that for photons, with two important differences. First, the volume *V* is replaced by the SPP-field volume *V*_SPP_=*A*·*L*_*z*_, where *A* is the metal–dielectric interface area and *L*_z_ the decay length of the SPP in the metal. Since *L*_*z*_ is of order 10 nm at visible wavelength for noble metals^1^ and thus much shorter than the light absorption depth, the electric field associated with the SPP is concentrated to a small volume *V*_SPP_ at the metal–dielectric interface^1^, resulting in enhanced local generation of HCs from SPPs compared with light. Second, the SPP wavevector **q**_p_ is in general comparable to the size of the BZ, while for photons **q**_p_≈0. The finite SPP wavevector can significantly alter the phase space available for SPP decay, as dictated by the **q**_p_ dependence of the FM-JDOS. We remark that additional momentum can be transferred by phonons and defects assisting the SPP scattering process.

For both SPP and photon decay, the FM-JDOS regulates the phase space for HC generation. Since the experimental conditions and the material-specific SPP dispersion relation dictate the SPP energy *E*_p_ and wavevector **q**_p_^1^,^30^,^31^, we study the FM-JDOS as a function of **q**_p_ and *E*_p_ and arbitrarily treat them for the purpose of this work as independent variables; HC generation from light is obtained as the specific limit **q**_p_=0. We note that the SPP decay rate Γ_p_(**q**_p_,*E*_p_) could also be computed by evaluating the coupling matrix elements explicitly for fixed experimental conditions and dispersion relation^1^ 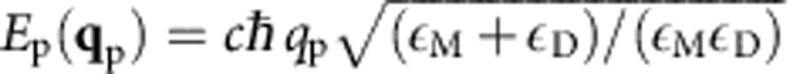. This approach would enable first-principles calculations of plasmonic losses and the MFPs of SPPs in materials^4^,^5^.

### HC generation

**Figure 1:**
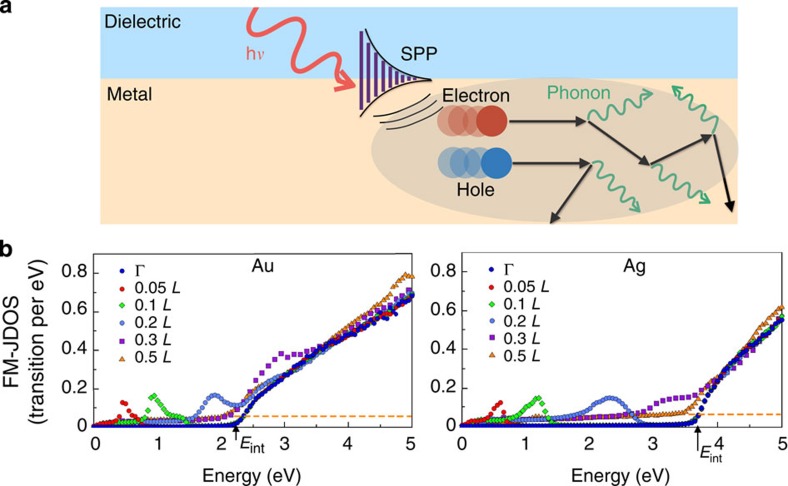
Hot carrier generation in Au and Ag. (**a**) Schematic of a metal–dielectric interface under illumination, showing the decay of a SPP to a pair of hot electron and hole. The hot carriers are scattered and lose energy through phonon emission processes. The mean free path corresponds to the length of the black arrows, and the excited volume within the metal is shaded in grey. (**b**) The FM-JDOS of Au (left panel) and Ag (right panel) as a function of SPP energy, shown for several values of **q**_p_ along the Γ–L direction, up to a value of **q**_p_=0.5 *L*. The SPP wavevector is expressed in units of the *L* point in the BZ, where *L*=(*π*/*a*)(1,1,1), with *a* the lattice constant. The FM-JDOS for photons and SPPs with vanishingly small wavevector corresponds to the **q**_p_=0 case (curve labelled as Γ). The dashed lines indicate the value of the FM-JDOS low-energy tail for large wavevectors.

To study HC generation from SPPs at a metal–dielectric interface (see Fig. 1a), we compute the FM-JDOS for Au and Ag, as shown in Fig. 1b, for SPP energies up to 5 eV and increasing SPP wavevectors along the Γ–*L* direction in the BZ (that is, for SPPs propagating along the [111] crystallographic direction). Our calculations are carried out using GW bandstructures (see Methods).

**Figure 2:**
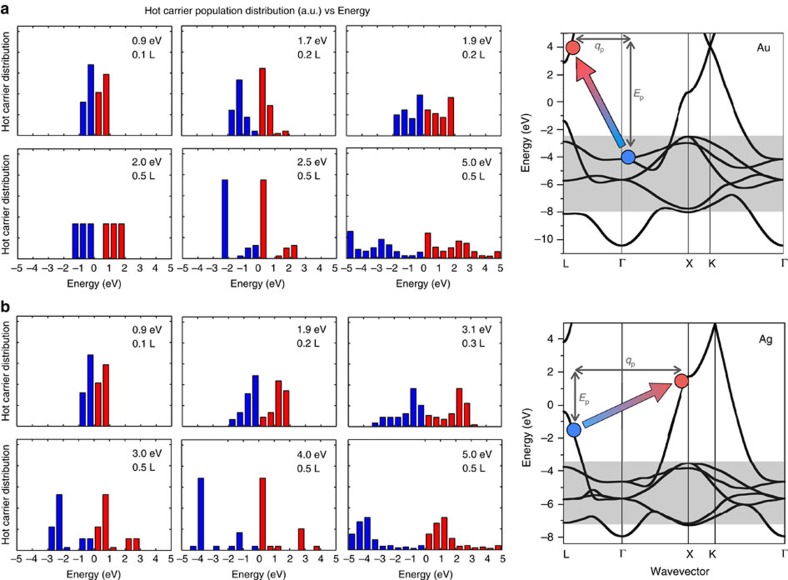
Energy distribution of hot carriers. Energy distribution of hot electrons (red) and hot holes (blue) in Au (**a**) and Ag (**b**) generated by SPP decay. Each plot refers to a specific SPP energy and wavevector. The right panel shows the GW bandstructures of Au and Ag, respectively, in **a** and **b**, along with a schematic of the momentum and energy conserving excitation of a hot electron–hole pair resulting from the decay of a SPP with wavevector **q**_p_ and energy *E*_p_. The shaded area indicates the energy range of the *d* states. The energy difference between the Fermi energy and the top of the *d* bands corresponds to the interband transition threshold. The Fermi energy is the zero of the energy axis.

Detailed features of the bandstructures of Au and Ag, shown in Fig. 2, regulate HC generation and transport in these materials. The main features of the Au and Ag bandstructures are the presence of relatively dispersionless, occupied *d* bands with large associated electronic DOS, straddled by a free-electron-like *s* band. The top of the *d* bands is located at energy *E*_int_ below the Fermi energy, where *E*_int_ is the threshold for interband (*d* to *s*) electronic transitions^32^,^33^. From our GW calculations, we find *E*_int_ values of 2.3 eV in Au and 3.7 eV in Ag (see Fig. 2), in excellent agreement with experiment^5^,^32^,^33^. The free-electron-like *s* band spans an energy window between *E*_int_ below and over 4 eV above *E*_F_, and hence dominates the electronic properties of Au and Ag near *E*_F_.

The FM-JDOS in Fig. 1b follows similar trends in Au and Ag, with deviations due to the different *E*_int_ values in the two materials. We first discuss the FM-JDOS with **q**_p_=0, which quantifies the phase space for HC generation for both SPPs with very small wavevectors and photons. The FM-JDOS curve with **q**_p_=0 (Γ) has a zero value for SPP energies up to *E*_int_, indicating the absence of possible SPP decay into HCs in this energy range. For SPP energies greater than *E*_int_, the FM-JDOS increases linearly with energy, indicating that a large phase space opens up for HC generation. In this regime, SPP damping is strong, and HC generation is intense and dominated by formation of hot *d* holes collecting most of the energy from the SPP, as discussed below.

For small SPP wavevectors along the Γ–*L* direction up to ∼0.2*L*, we find a peak in the FM-JDOS corresponding to SPP decay through intraband transitions within the *s* band. The energy position of the peak increases linearly with SPP wavevector, and for large enough wavevectors merges with the FM-JDOS above *E*_int_. In Au, the intraband peak is below *E*_int_ for **q**_p_ values up to ∼0.2 *L*, and in Ag for **q**_p_ values up to ∼0.3 *L* due to the higher *E*_int_ value in Ag. The presence of an intraband peak associated with *s*–*s* transitions can be understood by examining the energy difference between states with momentum **k** and **k**+**q**_p_ in the free-electron-like *s* band:


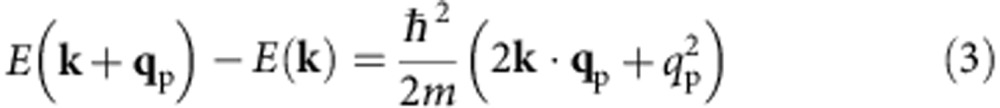


For small wavevector **q**_p_, the term quadratic in *q*_p_ can be neglected, while the term linear in **q**_**p**_ increases by ∼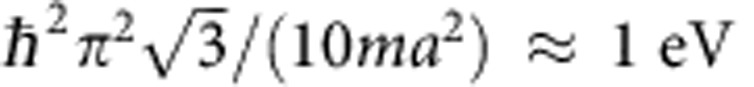 (*a* is the lattice constant) for an increase of **q**_p_ by 1/10 the Γ–*L* distance. The computed FM-JDOS confirms these trends, as seen by the increase in the energy of the intraband peak by ∼1 eV in going from 0.1 *L* to 0.2 *L* and from 0.2 *L* to 0.3 *L*. In this small wavevector regime, generation of HCs is appreciable only if the SPP energy and wavevector are matched to the intraband FM-JDOS peak, namely if *E*_p_(**q**_p_)≈(*ℏ*^2^**k**·**q**_p_)/*m* for a set of **k** points in the BZ. When this condition is met, intense HC generation from intraband transitions can occur, resulting in an almost equal apportioning of the SPP energy between hot electrons and holes, as discussed below.

For SPP wavevectors larger than ∼0.3 *L*, the FM-JDOS resembles that for **q**_p_=0 except from a flat tail that extends to low SPP energy, as shown by the dashed lines in Fig. 1. This tail is due to generation of HCs from intraband transitions with large transferred momentum from the decay of SPPs with wavevector comparable to the size of the BZ. The FM-JDOS for **q**_p_>0.5 *L* is unchanged and nearly identical to the 0.5 *L* case.

To summarize, we find two HC generation regimes. For SPPs with energy *E*_p_<*E*_int_, optimal generation can be achieved using SPPs with relatively small wavevector (less than ∼1/5 the BZ size) that are matched with the FM-JDOS peak, while HC generation is relatively weak for SPPs with larger momentum. For SPPs with energy *E*_p_>*E*_int_, HC generation is intense regardless of the SPP momentum. We find identical trends for SPPs with wavevector in the Γ–X high-symmetry direction, that is, propagating along the [100] direction. Removal of the electron gas approximation used in simplified treatments of SPPs in noble metals^17^ is necessary to capture the delicate interplay found here between the energetics of the *s* and *d* states and intraband (*s*–*s*) and interband (*d*–*s*) transitions.

### Energy distribution of HCs

Figure 2 shows the energy distribution of HCs generated by specifically assumed SPPs with energy increasing in small steps from ∼1 to 5 eV and a range of wavevectors in the Γ–*L* direction, along with the GW bandstructures. From now on throughout the manuscript, all HC energies are referenced to the Fermi energy (*E*_F_), and the energy of the hot hole is −*E*, that is, the direction of increasing energy in the bandstructure is downward for holes and upward for electrons. The energy distribution histograms identify the HCs generated in the two regimes discussed above. For SPPs with energy lower than *E*_int_, hot holes and electrons are generated with a roughly uniform energy distribution as a result of the *s*–*s* intraband transitions. The HC energy distributions shown in Fig. 2 in this energy range pertain to SPPs with energy and wavevectors matched to the FM-JDOS intraband peaks (see Fig. 1). While the details of the energy distribution in this regime are sensitive to the SPP energy *E*_p_, the overall trends indicate that HCs are generated with an average energy of ∼*E*_p_/2.

For SPPs with energy greater than *E*_int_, we find a change to a different HC generation regime. At the onset of the interband transitions for SPP energy of 2.3 eV in Au and 3.7 eV in Ag, on SPP decay the hot holes absorb most of the SPP energy, creating a spike in the hot hole population at the top of the *d* band. On the other hand, hot electrons have only modest energies in this regime. At higher SPP energies of ∼5 eV, the HC population become approximately uniformly distributed over a wide energy range, although a peak in the hot *d* hole population is still present in Ag due to its higher *E*_int_. These trends are common to both Au and Ag, and we have verified that Cu, a material less commonly used in plasmonics^5^ and not discussed here, follows identical HC generation and energy distribution trends as Au and Ag.

### HC scattering processes

While ample experimental^13^,^32^ and theoretical^14^ data exists on HCs in noble metals, characterization of the MFPs and relaxation times of HCs using *ab initio* theory has been limited by the absence of accurate *e*–ph calculations including all phonon modes over the entire BZ. First-principles calculations combining *e*–ph and *e*–*e* interactions are necessary to characterize the anisotropic and energy-dependent MFPs of HCs in materials, as shown in our recent work on HCs in semiconductors^21^. We compute the scattering rate (and its inverse, the relaxation time) for the *e*–ph and *e*–*e* interactions from the imaginary part of the respective self-energies, 
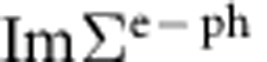
 and 
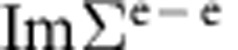
, and resolve these quantities for each electronic state with band *n* and **k**-point on very fine BZ grids (see Methods and ref. 21). The total relaxation times *τ*_*n*,**k**_, combining the *e*–ph and *e*–*e* interactions, are obtained as 
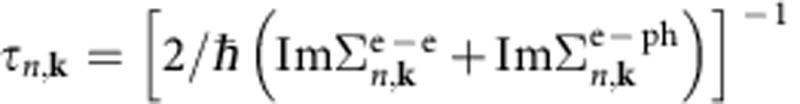
.

**Figure 3:**
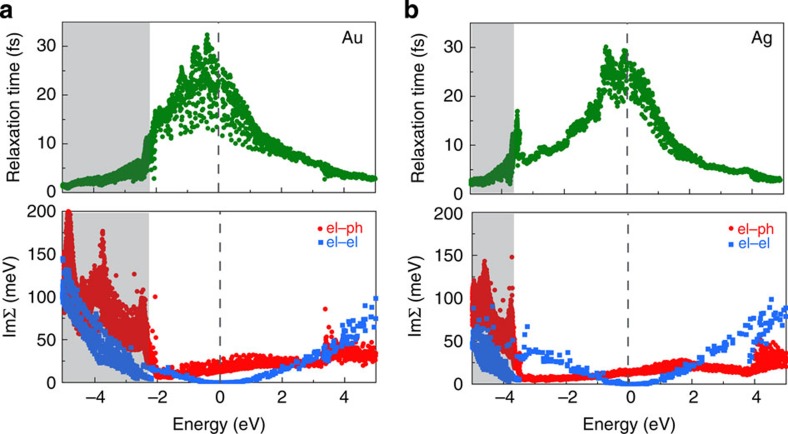
Ultrafast hot carrier scattering. Relaxation time and scattering rates for HCs in Au (**a**) and Ag (**b**) within 5 eV of the Fermi energy. The top panels show the total relaxation time, accounting for both the *e*–*e* and the *e*–ph interactions. The bottom panels show the scattering rate, expressed as the imaginary part of the self-energy, 
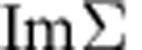
, for the *e*–*e* and *e*–ph interactions. *E*_F_ is the zero of the energy axis, as shown by the dashed line. Electrons (holes) possess positive (negative) energies, and the shaded area indicates the energy window spanned by the *d* states.

Figure 3 shows the total relaxation times *τ*_*n*,**k**_ for HCs in Au and Ag with energy up to 5 eV. The total relaxation times decay rapidly away from *E*_F_ following a volcano-shaped trend, with the peak of the volcano centred close to *E*_F_. In Ag, we find an additional peak in the relaxation times at the top of the *d* bands, in agreement with recent photoemission experiments^34^. Analysis of the *e*–ph and *e*–*e* scattering rates (see the bottom panels in Fig. 3) highlights the differences between the energy windows spanned by the *s* and *d* states, and elucidates the origin of the relaxation time trends. In the energy range spanned by the *s* states, the *e*–*e* scattering rates (that is, 
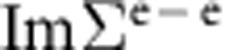
) form a parabola with minimum value of zero at *E*_F_ as predicted by Fermi liquid theory^14^, while the *e*–ph scattering rates are relatively constant and exhibit a minimum at the onset of the *d* states and a maximum 1–2 eV above *E*_F_. The *e*–*e* scattering rates become greater than the *e*–ph ∼2 eV away from *E*_F_, thus indicating that Auger and impact ionization processes included in the *e*–*e* term dominate HC scattering only 2 eV or more away from *E*_F_, while *e*–ph dominates HC scattering within 2 eV of *E*_F_. Combining the two scattering mechanisms leads to total relaxation times with a broad maximum centred at *E*_F_ and a rapid decay 1–2 eV away from *E*_F_ as the parabolic *e*–*e* scattering rate becomes dominant (see Fig. 3). In the energy window spanned by the *d* bands, the large increase in the electronic DOS causes very strong *e*–ph scattering, and the localized nature of the *d* states leads to deviations from the free-electron parabolic trends for *e*–*e* scattering. The total relaxation times show that hot holes arising from *d* states lose energy on a sub-5-fs time scale, thus making such hot *d* holes challenging to extract before thermalization. Finally, our calculations yield comparable time scale and scattering rate for *e*–ph and *e*–*e* scattering, in contrast with the common heuristic assumption that scattering by phonons occurs on a much slower time scale^14^. We thus conclude that previous models including only *e*–*e* scattering^14^ and missing the important *e*–ph component are incomplete for understanding HC relaxation times and photoemission linewidths in noble metals.

To validate our results, we compare our relaxation times at the Fermi energy in Au and Ag with those inferred from carrier transport measurements. Kopitzki^35^ combined the Drude model with room temperature resistivity measurements, and obtained semiempirical relaxation times of 29 fs for Au and 41 fs for Ag, in close agreement with those obtained by Ashcroft and Mermin^36^ with the same approach. Averaging our relaxation times over a small energy window around *E*_F_ yields relaxation times of 24 fs for Au and 27 fs for Ag. While the agreement is excellent for Au and good for Ag, our data shows a large spread in the relaxation times at *E*_F_, a feature not captured by the Drude model and stemming from the Fermi surface anisotropy. It is therefore puzzling that the Drude relaxation times^33^,^35^ are widely used in plasmonics^4^,^5^, given that their physical meaning is questionable when applied to non-equilibrium situations involving SPPs. We conclude that further investigation on this point is necessary, and remark that ours is the first truly *ab initio* determination of the relaxation times on the Fermi surface of noble metals, to be discussed in detail elsewhere.

### HC MFP

**Figure 4:**
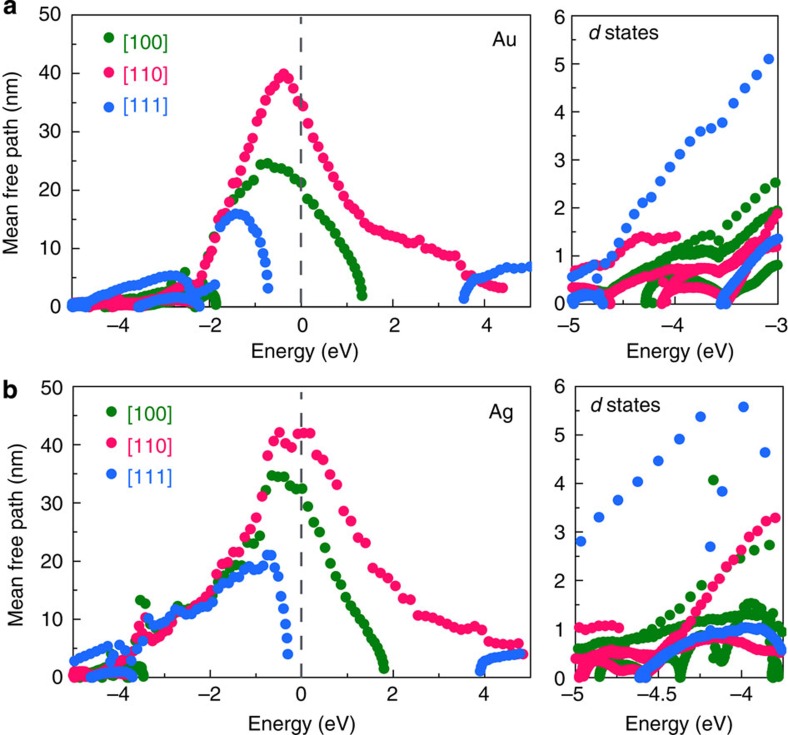
Hot carrier mean free path. The hot carrier MFPs along the [100], [110] and [111] crystallographic directions in Au (**a**) and Ag (**b**). The MFPs for the *d* states are expanded in the right panels.

Next, we turn our attention to HC transport. Figure 4 shows the MFP for the three crystallographic directions [100], [110] and [111] within 5 eV of the Fermi energy. The MFPs are obtained by multiplying the total relaxation times by the carrier velocities computed from the slope of the GW bandstructures (see Methods). Our computed MFPs are of the order 10–40 nm in the energy window spanned by the *s* states, and much shorter (1–5 nm) for the *d* states. The MFPs of the *s* states exhibit an energy dependence with the volcano shape also seen in the relaxation times, with deviations coming from the different band velocities along each direction. The MFPs of hot holes with 1–2 eV energy are nearly isotropic, while the MFPs of hot holes with <1 eV energy are longer for the [100] and [110] directions. The MFPs of hot electrons are highly anisotropic due to the absence of electronic states up to 4 eV above *E*_F_ in the [111] direction and at energy >1–2 eV in the [100] direction. The longest hot electron MFPs are in the [110] direction up to 4 eV above *E*_F_. We note that the energy derivative of the MFPs at *E*_F_ at is negative, consistent with the positive thermopower in Au and Ag^37^. In the energy window spanned by the *d* states, the MFPs are of order 5 nm in the [111] direction and 1 nm in the other directions, and thus highly anisotropic with longer MFPs in the [111] direction. The isotropic MFPs for *s* holes and anisotropic for *d* holes are consistent with findings in ballistic electron energy microscopy experiments in noble metals, where it was found that *d* holes injected by a scanning tunneling microscope tip propagate along narrow cones and span small volumes, while *s* holes span large volumes isotropically^38^.

## Discussion

**Figure 5:**
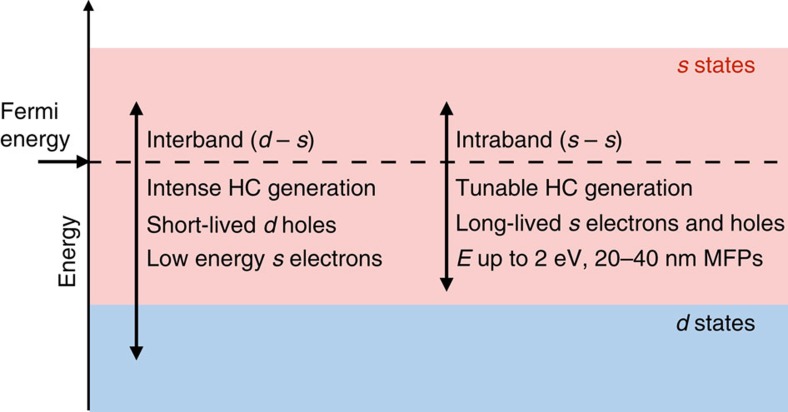
Regimes for hot carrier generation and transport in noble metals. The graph shows two HC generation regimes, for intraband (*s*–*s*) and interband (*d*–*s*) transitions induced by SPPs with energy *E*_p_<*E*_int_ or *E*_p_>*E*_int_, respectively. The regime with *E*_p_<*E*_int_ offers a trade-off between optimal HC generation, moderate HC energies up to ∼2 eV, and longer MFPs and relaxation times than the interband regime. The dashed line indicates the Fermi energy.

The data obtained so far allow us to formulate optimal conditions to generate HCs and extract or utilize them before thermalization. Figure 5 summarizes HC generation and transport in Au and Ag by defining two regimes for HC generation from intraband (*E*_p_<*E*_int_) and interband (*E*_p_>*E*_int_) transitions induced by SPP decay. The energy distribution, relaxation time and MFP of the generated HCs are dramatically different in these two regimes. For *E*_p_<*E*_int_, the HCs consist of *s* holes and *s* electrons with average energies up to *E*_int_/2 (that is, 1–2 eV) generated by weakly damped SPPs due to the small FM-JDOS values. HC generation can be optimized by matching the energy and momentum of the SPP to the FM-JDOS intraband peak described above and shown in Fig. 1. The HCs in this energy range possess relatively long MFPs of ∼10–40 nm, and can thus be extracted before thermalization over ∼50 nm lengths in ideally pure samples. This regime is thus optimal for HC generation and extraction. For hot electrons, we predict that the (110) surface would be best suited to extract HCs due to the longer MFPs in the [110] direction. For hot holes with ∼1–2 eV energy, the (100) and (110) surfaces are predicted to enable optimal extraction. Our results further suggest that Ag may be better suited than Au for HC generation due to the wider energy window for intraband transitions, thus motivating studies of HC generation from SPPs in Ag. On the other hand, the strong SPP damping regime with *E*_p_>*E*_int_ is non-optimal for extracting HCs, since in spite of strong HC generation the SPP energy is mostly transferred to short-lived hot *d* holes with ∼1 nm MFP, while hot electrons possess only low energy of <1 eV. These conditions prevent HC extraction in this regime, unless a nanometer-thick thin film or nanostructured metal is employed.

Our data further show that nanostructuring the metal to sub-10-nm size is not required to obtain energetic HCs, and not needed to extract them unless HCs with >2 eV energy are desired. In addition, for SPP with energy lower than *E*_int_, the SPP energy is equally distributed between hot electrons and holes, and energy loss is dominated by *e*–ph rather than Auger scattering. These findings address the misconceptions emerged in recent work as outlined above, and show that optimal HC generation is possible by carefully tuning the SPP energy and wavevector at noble metal–dielectric interfaces. Finally, we note that our approach is for now limited to SPP at metal–dielectric interfaces, while localized surface plasmons in nanostructures need to be treated differently due to their localized nature^39^. We believe that the method presented in this work can be extended to study HC generation from localized surface plasmon modes, which will be the subject of future investigation.

In summary, we establish a theoretical framework to study SPP damping and HC generation and transport on the same footing using many-body perturbation theory. Our first-principles calculations can accurately describe SPP-induced generation and ultrafast scattering of HCs in noble metals of use in plasmonics, photocatalysis, photovoltaics and optoelectronics. Our work highlights the interplay of the *s* and *d* bands in noble metals, and prescribe optimal experimental conditions for generation and extraction of HCs. Our approach paves the way to first-principles calculations of SPP losses in materials^4^.

## Methods

### First-principles calculations

We carry out *ab initio* calculations on Au, Ag and Cu in the face-centred cubic structure with lattice parameters of 7.72, 7.71 and 6.82 bohr, respectively. The ground-state electronic structure is computed within the local density approximation (LDA)^40^ of DFT using the QUANTUM ESPRESSO code^41^. We use norm-conserving pseudopotentials (which include semicore *s* and *p* states) to describe the core-valence interaction^42^, together with a plane-wave basis set with kinetic energy cutoff of 60 Ry for Ag and Au, and 240 Ry for Cu. The *e*–*e* (that is, GW) and *e*–ph self-energies are computed separately, and then combined together.

The real and imaginary parts of the GW self-energy^19^ are computed using the Berkeley-GW code^43^. The real part 
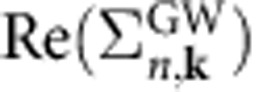
 is obtained with a generalized plasmon–pole calculation^19^ on a 12 × 12 × 12 **k**-point grid and then interpolated using Wannier functions (see below). Kinetic energy cutoffs of 50 Ry and 120 Ry are used, respectively, for the screened and bare Coulomb interactions, together with ∼1,000 empty bands and a 8 × 8 × 8 **q**-point grid for the dielectric screening. The DFT eigenvalues 
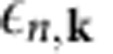
 are corrected with GW self-energies to obtain the quasiparticle bandstructures, 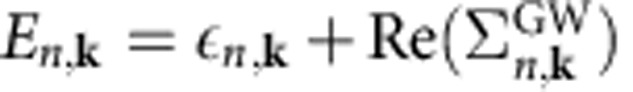, used in all calculations in this work. The imaginary part of the GW self-energy, 
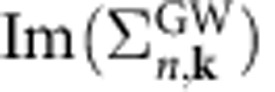
, is computed using full-frequency GW calculations^43^. Here 
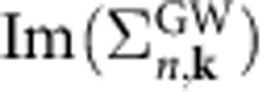
 are evaluated on-shell at the LDA eigenvalues 
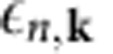
, and then plotted versus the corresponding GW eigenvalues *E*_*n*,**k**_. We use 20 × 20 × 20 **k**-point grids to converge 
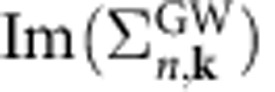
 within ∼10 meV. In addition, we use kinetic energy cutoffs of 20 Ry and 120 Ry, respectively, for the screened and bare Coulomb interactions, together with ∼100 empty bands and a 20 × 20 × 20 **q**-point grid for the dielectric screening.

The imaginary part of the *e*–ph self-energy, 
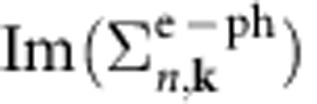
, is computed using the EPW code^20^. We compute the self-consistent potential and Kohn–Sham states on a 12 × 12 × 12 **k**-point grid using DFT, and lattice-dynamical properties with DFPT^44^ on a 4 × 4 × 4 **q**-point grid. The *e*–ph matrix elements are first computed on these coarse grids, and then obtained on significantly finer grids using an interpolation procedure based on Wannier functions as implemented in the EPW code^20^,^24^. Maximally localized Wannier functions^45^ are obtained starting from five *d* orbitals on the metal atoms and two *s* orbitals, each in a tetrahedral interstitial point of the face-centred cubic lattice, for a total of seven wannierized bands (we skip the semicore states). The Wannier interpolated and DFT bandstructures agree within 5 meV in an energy window up to 5 eV above the Fermi energy. The Bloch-to-Wannier rotation matrices are then used to interpolate the GW bandstructures, which are used to compute 
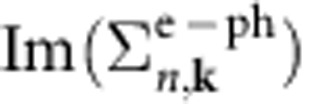
. The fine grids for the calculation of 
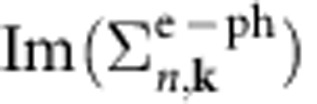
 consist in a 40 × 40 × 40 **k**-point grid, and up to 1 million random phonon **q**-points in the BZ. Such fine grids allow us to converge 
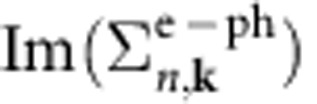
 within 1 meV. Further details of our approach to compute the *e*–ph self-energy are discussed in ref. 21.

The FM-JDOS calculations are carried out using Wannier interpolated GW bandstructures, a 100 × 100 × 100 **k**-point grid and a small Lorentzian broadening *η*≈25 meV. The energy distribution of the generated HCs in Fig. 2 are obtained by counting the momentum- and energy-conserving transitions in equation (2) as a function of occupied band (for hot holes) and empty band (for hot electrons) quasiparticle energies.

The MFPs are obtained using velocities computed from the slope of the GW bandstructure along the given high-symmetry direction, together with total relaxation times *τ*_*n*,**k**_ combining the *e*–ph and *e*–*e* relaxation times with Matthiessen's rule. The total relaxation times are obtained from 
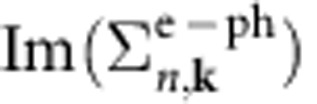
 computed on grids of over 100 **k** points along each high-symmetry direction, and 
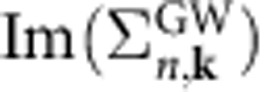
 computed on a 20 × 20 × 20 **k**-grid and then averaged over **k** points to generate an energy-dependent 
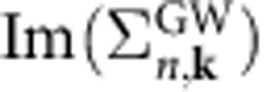
 evaluated at each energy *E*_*n*,**k**_ for which a calculation of 
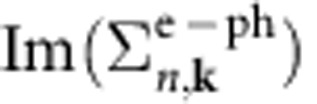
 is performed.

### Derivation of the coupling matrix elements

The derivation of the plasmon-electron matrix element in Table 1 within the Landau damping approximation is given in ref. 26 and will not be discussed. Here we derive the coupling matrix elements for the photon–electron and SPP–electron interactions given in Table 1. We use SI units throughout the derivations. The photon case is well known^27^ and discussed here as a starting point for the SPP case. The second quantized vector potential 
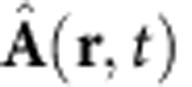
 for a photon with momentum *ħ***q**_p_, energy 
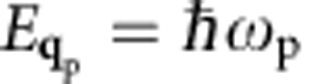
 and polarization unit vector 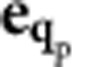 can be obtained by associating the amplitude with a destruction operator 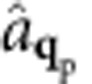 and its complex conjugate with a creation operator 
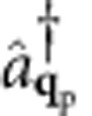
. We use the vector potential with unit amplitude since we are interested in the coupling matrix element, and thus the transition rate per unit of incident power. It reads^27^:


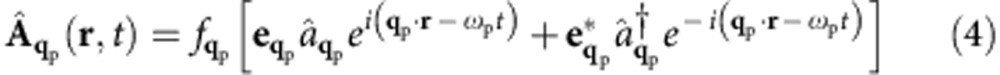


where 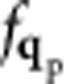 is a normalization constant. Using the Coulomb gauge 
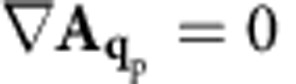
, the second quantized electric field can be obtained from 
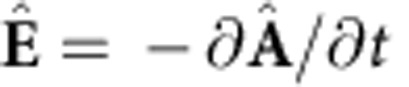
.

The normalization constant 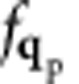 is determined by equating the energy of the classical and quantized fields. The classical (cycle averaged) energy 
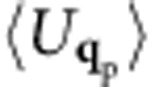
 for a photon with momentum *ħ***q**_p_ in a homogeneous and isotropic material with dielectric constant 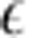 is 
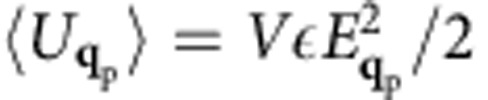
, where *V* is the volume of the material. This energy needs to be equal to its quantum counterpart, 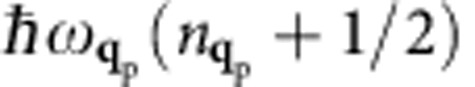. In addition, 
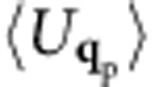
 can be obtained by averaging the quantized electric field 
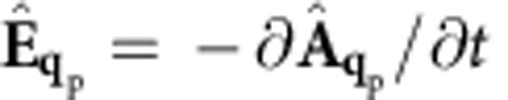
 over the quantum state 
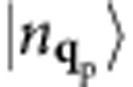
 with 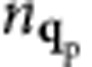 photons. We get:


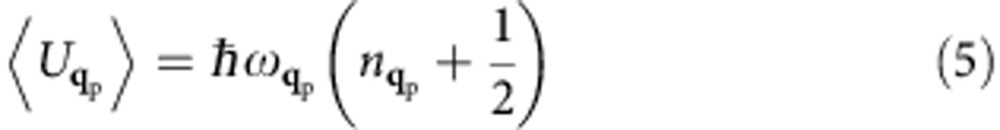



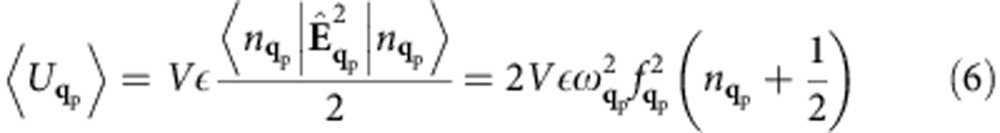


where in the last equation we used the time derivative of equation (4) the electric field and the commutation rules for boson creation and annihilation operators, 
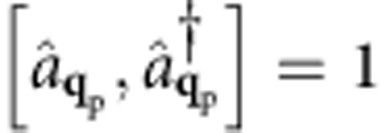
. Equating equations (5) and (6) yields a normalization constant 
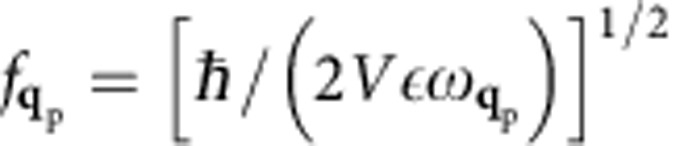
, and a vector potential:


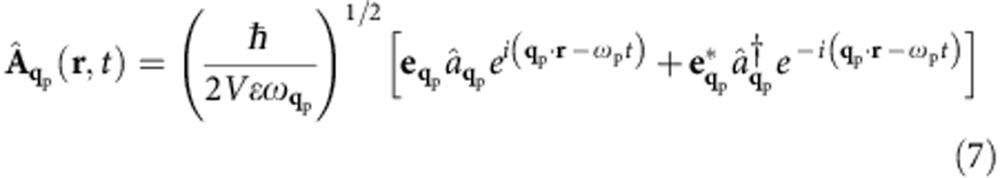


The photon–electron interaction Hamiltonian is 
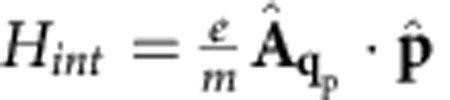
, as obtained by expanding the kinetic energy 
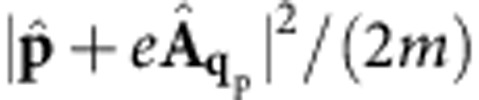
 to first order in the vector potential. The second quantized fields associated with the electronic Bloch states are:


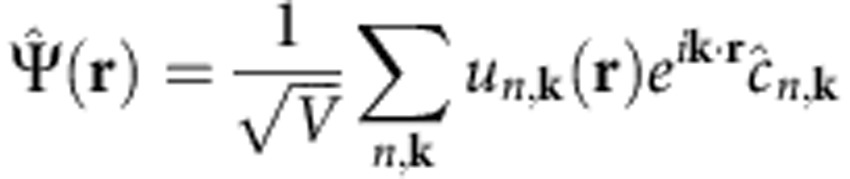



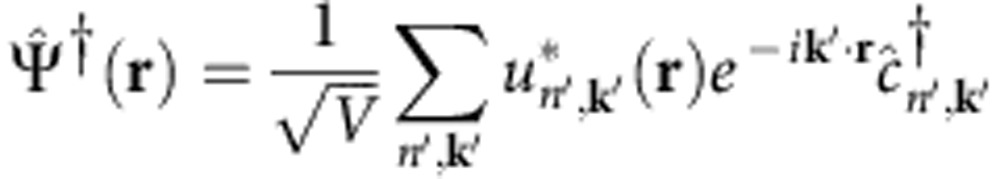


where 
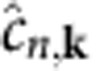
 and 
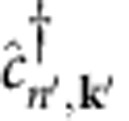
 are, respectively, fermion annihilation and creation operators satisfying the anticommutation rule 
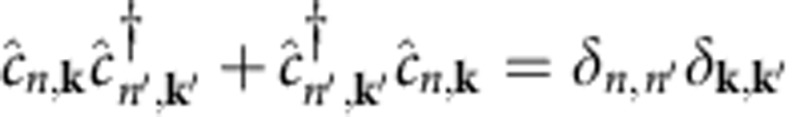
. The second quantized interaction Hamiltonian can be written as:


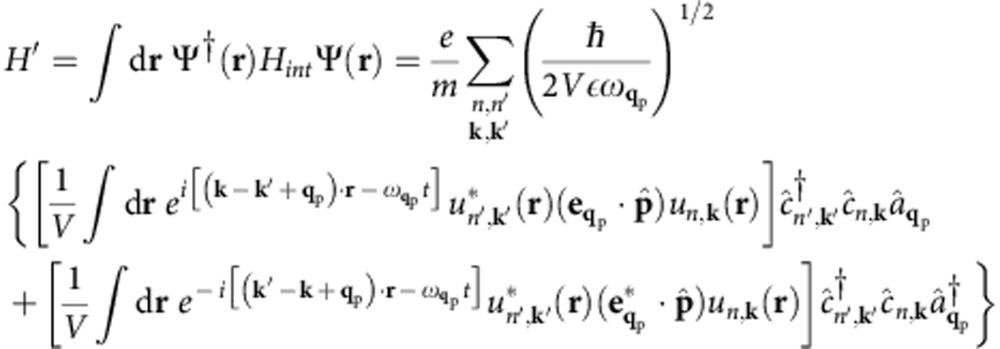


Crystal momentum conservation requires **k**′=±**q**_p_, where the plus and minus sign apply for the first and second integral above, respectively. The interacting Hamiltonian becomes:


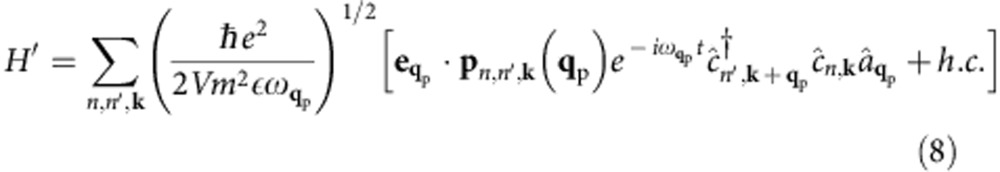


where *h.c.* is the hermitian conjugate, and we defined the momentum matrix element as the integral 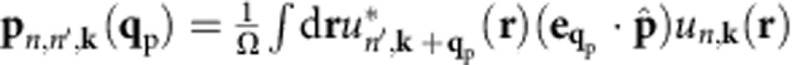 over the unit cell volume Ω, as commonly used in the study of optical absorption^46^. We obtain the photon decay rate 
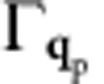
 by applying Fermi's golden rule. Photon absorption occurs with a transition rate 
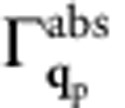
 between an initial state 
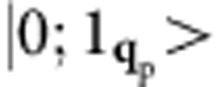
, consisting of the ground electronic state at room temperature and one photon with momentum **q**_p_, and a final state |*S*;0〉 with an electron in the Bloch state |*n*′,**k**+**q**_p_〉, a hole in the Bloch state |*n*,**k**〉 and no photons. The absorption rate is given by:


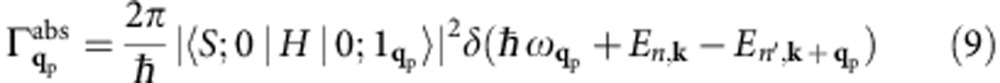


Only the first term in equation (8) gives a non-zero contribution, and we get:


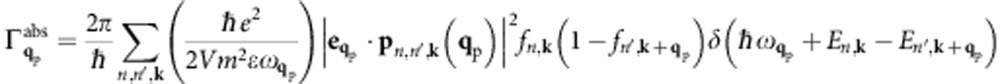


The inverse process of photon emission from an initial state |*S*;0〉 to a final state 
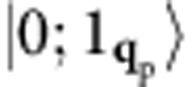
 has a rate 
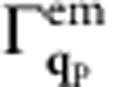
 given by:


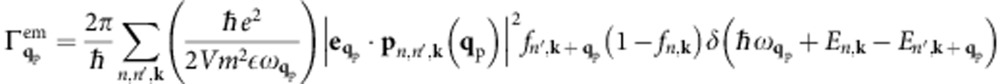


The net rate 
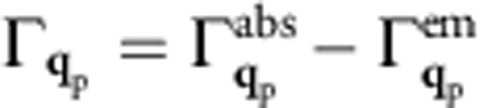
 is the decay rate of a photon with momentum *ħ***q**_p_ and energy 
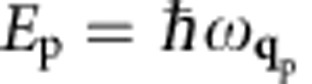
 into a hot electron–hole pair. Writing the delta function as a Lorentzian with broadening *η*→0, namely 
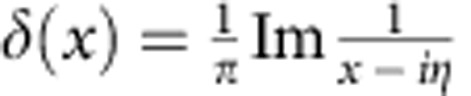
, we obtain the decay rate as:


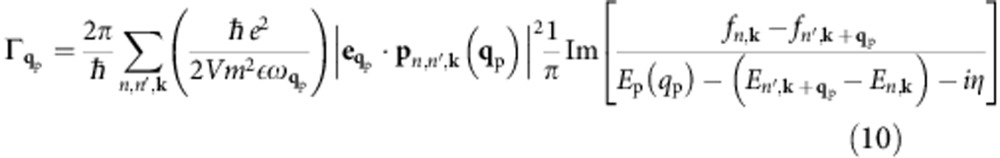


This expression is identical to equation (1) with the squared coupling matrix elements 
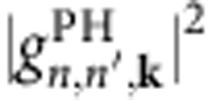
 in Table 1. Since for photons **q**_p_≈0 compared with the size of the BZ, the squared coupling matrix elements are:


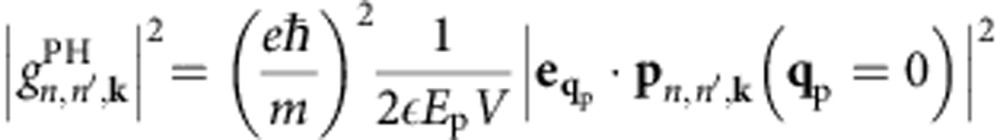


as shown in Table 1. We note that for slowly varying matrix elements in the BZ, the photon decay rate is proportional to the zero-momentum JDOS commonly used in the theory of the optical properties of solids 46, that is, 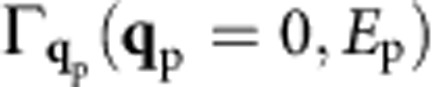.

The derivation of the coupling matrix elements 
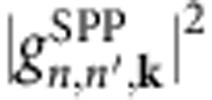
 for SPPs follows similar steps to the photon case discussed above. Following Nkoma *et al*.^28^, we study the case of a SPP generated at the interface between a metal with frequency-dependent dielectric function 
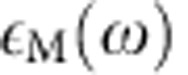
 and a dielectric material with frequency-independent dielectric constant 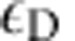. A similar second quantization procedure was also developed by Elson *et al*.^29^. It is well known that the intensity of the SPP wave decays exponentially in the direction perpendicular to the dielectric-metal interface^1^,^30^. To study HC generation, we focus here on the SPP field in the metal, and its associated vector potential. Since SPP possess transverse magnetic polarization, their wavevector can be taken as real in the *xy* plane—here the plane of the metal–dielectric interface, with area *A*—and purely imaginary in the *z* direction normal to the interface. With the metal in the *z*>0 space and the *x*-axis parallel to the propagation direction of the SPP wave, the SPP wavevector is **q**_p_=(*q*_p,*x*_,0,*iq*_p,z_). While in the main text, we define **q**_p_=(*q*_p,*x*_,0,0) as the propagation wavevector in the *xy* plane, for the purpose of this derivation **q**_p_ is defined differently. Here *q*_p,*x*_ is a real positive number since the electron-induced dissipation effects we aim to find would be encoded in its imaginary part, and *q*_p,*z*_ is related to the decay length *L*_z_ of the SPP field intensity by^30^ *q*_p,*z*_=1/(2*L*_*z*_). The relative magnitude of the components of **q**_p_ are determined by the dielectric properties of the metal and dielectric^1^,^30^, which impose 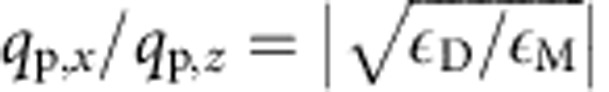 (here, the real parts of 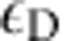 and 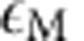 should be employed for consistency). Similarly, the components of the unit polarization vector 
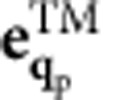
 are related by^30^ 
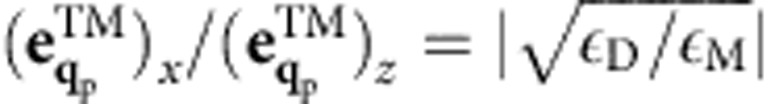
.

The vector potential of the SPP wave in the metal, with unit amplitude as in the photon case, can be written in second quantized form as:


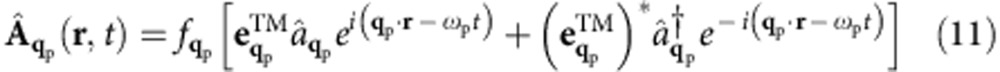


where, as in the photon case, the normalization constant 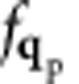 can be determined by energy considerations. We write the classical electric field associated with the SPP in the metal as 
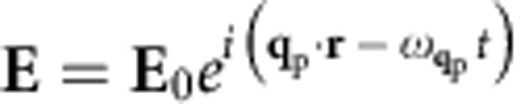
. Using a result in ref. 28, the cycle-averaged energy in the SPP field—which accounts for contributions from both the field in the dielectric and the metal—can be written as 
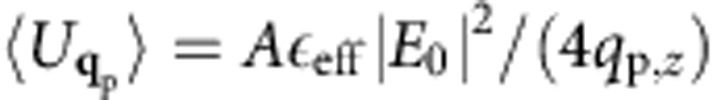
, where we defined an effective dielectric constant: 
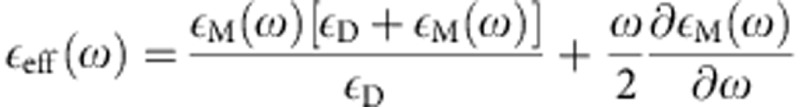


We use the correspondence principle for a quantum state 
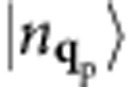
 with 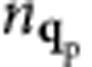 SPP quanta. Similar to the photon case, the total energy in the SPP mode 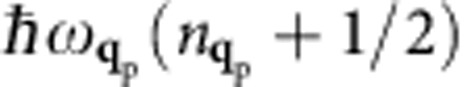 can be equated to its classical counterpart, while the square modulus of the electric field 
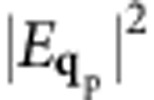
 can be replaced with its quantum mechanical average 
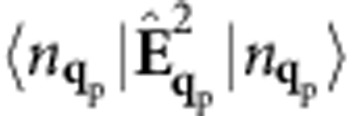
, with 
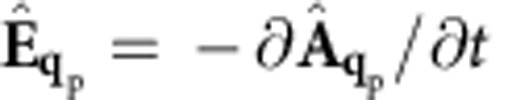
. We obtain the two equations:


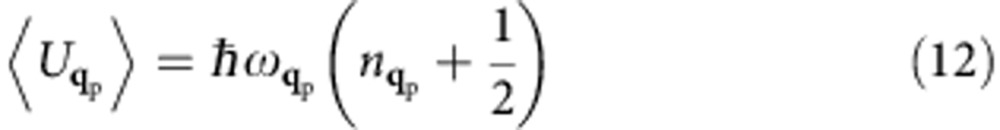



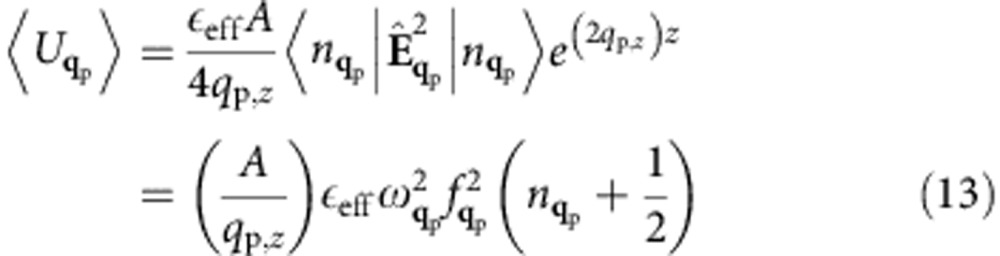


By equating equations (12) and (13), and using *q*_p,*z*_=1/(2*L*_*z*_), we obtain the normalization constant 
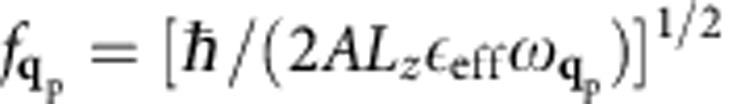
, and thus the SPP vector potential:


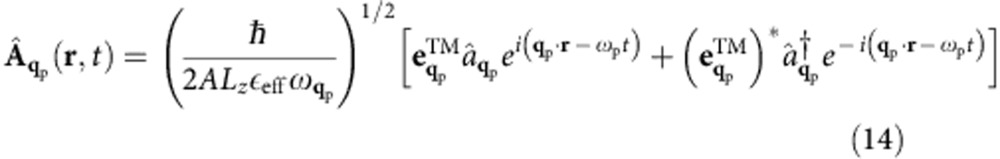


This vector potential is similar to the photon case, equation (7). The key differences are the decay in the *z* direction for the SPP field, which introduces an effective SPP volume *AL*_*z*_ in the vector potential, the appearance of an effective dielectric constant for the medium and the fact that the polarization is transverse magnetic with a direction imposed by the properties of the metal and dielectric material.

Since the form of the SPP vector potential is completely analogous to the one for photons, the decay rate of SPPs to hot electron–hole pairs can be carried out following the same steps as in the photon case discussed above, with the substitutions *V*→*AL*_*z*_, 
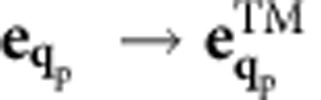
, and allowing for a finite momentum^1^ **q**_p_ with magnitude 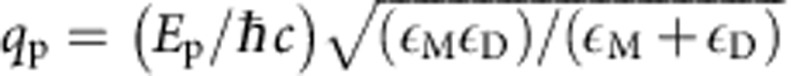. This leads to the SPP–electron coupling matrix element in Table 1:


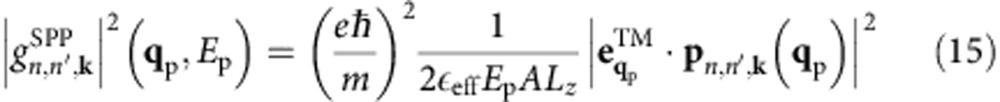


## Additional information

**How to cite this article:** Bernardi, M. *et al*. Theory and computation of hot carriers generated by surface plasmon polaritons in noble metals. *Nat. Commun.* 6:7044 doi: 10.1038/ncomms8044 (2015).

## Figures and Tables

**Table 1:** Boson–electron coupling and matrix elements.

**Figure:**